# Minimum Dietary Diversity for Children aged 6–23 months as a predictor of micronutrient adequacy in Ethiopia: Validation of the proxy indicator

**DOI:** 10.1371/journal.pone.0334827

**Published:** 2025-10-17

**Authors:** Yonatan Menber, Tefera Belachew, Netsanet Fentahun

**Affiliations:** 1 Department of Nutrition and Dietetics, School of Public Health, College of Medicine and Health Sciences, Bahir Dar University, Bahir Dar, Ethiopia; 2 Department of Nutrition and Dietetics, Faculty of Public Health, Institute of Health, Jimma University, Jimma, Ethiopia; Arizona State University, UNITED STATES OF AMERICA

## Abstract

**Background:**

The Minimum Dietary Diversity for Children aged 6–23 months (MDD-C) is a proxy indicator of micronutrient adequacy. There is a lack of evidence regarding the performance of this proxy indicator, including in Ethiopia, a country with diverse dietary habits. Therefore, this study aimed to validate the performance of MDD-C to predict micronutrient adequacy against the Mean Adequacy Ratio (MAR) among children aged 6–23 months in the North Mecha District, Northwest Ethiopia.

**Methods:**

A community-based cross-sectional study was conducted among 457 randomly selected study participants from February 2–18, 2023. A single multiphasic interactive 24-hour dietary recall was used to collect dietary intake data. Spearman’s rank correlation test, Cohen’s kappa statistics, and Receiver Operating Characteristic Curve analysis were conducted. The optimal cutoff point for MDD-C was determined by selecting the points that maximized the Youden index. Statistical significance was determined with a p-value < 0.05 using a 95% confidence interval.

**Results:**

The MDD-C had a moderate positive correlation (ρ = 0.62, p < 0.001) and a fair predictive ability (AUC = 0.78, 95% CI: 0.74, 0.83, p < 0.001) with the MAR to predict the micronutrient adequacy. The MDD score (≥5 food groups) had a sensitivity of 22.4% and a specificity of 91.4%, denoting slight agreement. The optimal cutoff point for MDD-C was found to be ≥ 3 food groups, with a sensitivity of 85.5% and a specificity of 62.9%. This optimal cutoff adjustment improved the agreement between the MDD-C indicator and micronutrient adequacy to a moderate level.

**Conclusions:**

The MDD-C had a moderate positive correlation and a fair predictive ability with the MAR to accurately predict micronutrient intake adequacy at the optimal cutoff, despite poor sensitivity and high specificity at the standard cutoff. Countries need to adapt MDD-C cutoffs to local dietary patterns to improve accuracy. WHO and UNICEF should revisit the global application of a uniform cutoff and support the development of context-specific thresholds. This study in Ethiopia underscores the importance of further research to reassess the effectiveness of MDD-C as a proxy indicator for determining micronutrient adequacy across diverse populations and multiple countries.

## Introduction

Early childhood is the most sensitive and vital window of life, during which individuals have a unique opportunity that is highly significant for cognitive, physical, social, and emotional development. The nutrition they obtain at this critical time ensures the required growth and development, as well as future health and productivity [1,2]. Inadequacies in micronutrient intake affect a considerable number of children in many countries [3–9], and malnutrition continues to constitute an important public health problem worldwide [10]. This concern is also observed in the case of Ethiopian infants and young children, where a significant proportion of children are affected by inadequate dietary diversity and its associated forms of malnutrition [11]. Enhancing Infant and Young Child Feeding (IYCF) practices is therefore a crucial and fundamental aspect of nutrition interventions [12].

There are different types of dietary assessment methods and indicators that are used to assess the nutritional adequacy of individuals and populations. Some of these measures, particularly quantitative methods and indicators, have a high degree of validity for accurately estimating nutrient intake. Still, they can be expensive, require skilled enumerators, and require a substantial time commitment for the assessment process. Therefore, there may be challenges in using these methods in large-scale surveys and implementing them regularly in resource-limited settings [13,14].

Among the available methods, the 24-hour Dietary Recall (24HR) is widely used for assessing dietary intake. Additionally, several indicators are used to measure dietary intake, including qualitative measures such as the Minimum Dietary Diversity for Children aged 6–23 months (MDD-C), along with quantitative measures like the Nutrient Adequacy Ratio (NAR) and the Mean Adequacy Ratio (MAR), which provide distinct strengths for evaluating dietary adequacy [13–16].

The NAR and MAR are used for assessing nutrient adequacy by analyzing quantitative dietary data. The NAR is a valuable tool that measures an individual’s nutrient intake against the Recommended Daily Allowance (RDA) to evaluate their nutritional adequacy and determine if they are meeting their daily nutrient requirements. On the other hand, the MAR serves as a holistic indicator of the overall nutritional quality of an individual’s diet. Despite its precision, it is difficult to use consistently [13]. As a result, another simple and inexpensive option should be considered a proxy indicator, such as Minimum Dietary Diversity (MDD) [12,17].

In 2021, the World Health Organization (WHO) and the United Nations Children’s Fund (UNICEF) revised the previous dietary diversity assessment and developed a new MDD indicator for children aged 6–23 months as one of the IYCF indicators. The new MDD is calculated based on eight food groups, which now include breast milk as the eighth food group. The minimum cutoff was increased to five food groups, following the 2008 guidelines that previously endorsed a 7-food-group MDD indicator and a cutoff of four food groups. The MDD-Cis one of the complementary feeding indicators used to assess IYCF practices [12,17]. The MDD-C, a qualitative dietary measure and an IYCF population-level indicator, can be used to conduct large-scale surveys, track progress toward national and international goals, assess the effectiveness of interventions, and make policy decisions, thereby saving resources due to its simplicity [12,17,18].

Several studies have assessed the performance of a 7-food group MDD in predicting micronutrient adequacy and have shown that adequate MDD predicts the adequacy of micronutrients [19–21]. A study was undertaken in the Philippines to evaluate the validity of the new 8-food group MDD-C in assessing nutrient adequacy [22]. These studies revealed that dietary diversity predicts the adequacy of micronutrient intake. There is a lack of further proof about the performance of the new MDD-C to predict micronutrient adequacy in multiple countries, including Ethiopia, where dietary consumption habits vary significantly among diverse communities. Therefore, this study aimed to validate the performance of the MDD-C as a predictor of micronutrient intake adequacy among children aged 6–23 months in the North Mecha District, Northwest Ethiopia.

## Materials and methods

### Study setting and study design

The study was carried out in the North Mecha District of the Amhara Regional State, which is located in Northwest Ethiopia, 530 kilometers from Ethiopia’s capital city, Addis Ababa. Agriculture plays a significant role in the district, as it provides food for 85% of the local population. The district is well known for its notable agricultural cultivation of teff, maize, barley, wheat, beans, and peas. Farmers in the district use a combination of rainfall and irrigation techniques to farm these crops [23]. The district houses the Koga Dam, which can irrigate 7,000 acres of land. The government initiated the Koga Irrigation and Watershed Management Project, which aims to improve agricultural output and water management in the region’s Koga watershed. This project aims to reduce poverty and improve food security. Its execution is expected to have a positive influence on food consumption and dietary patterns, as well as support the socioeconomic situation in the region [24]. As one of the more productive areas in the region, with year-round production, children in the district are expected to have a high level of dietary diversity and nutrient adequacy.

Since the primary aim of this study was to assess the discriminatory performance of the MDD-C against a reference measure (MAR) at a single point in time, and both indicators were derived from the same 24-hour recall data, a cross-sectional design was an efficient approach to achieve this evaluation objective without the need for follow-up. Accordingly, a community-based cross-sectional study design was employed from February 2^nd^ to 18^th^, 2023.

### Population and eligibility criteria

The source population for this study included children aged 6–23 months who lived in the North Mecha District. The study population consisted of children of the same age who resided in the district’s selected kebeles. Children who had lived in the selected study area for at least six months before the survey and who were living with their biological mother were included.

### Sample size determination and sampling techniques

A sensitivity estimation formula [25] was used to calculate the sample size using the following assumptions: a 95% confidence level, a 5% margin of error (d), a projected 90% sensitivity (Sn), and a 50% proportion (P). After multiplying by the design effect of 1.5 and adding a 10% nonresponse rate, the total sample size was 457. A multistage sampling technique was employed to select participants. First, seven kebeles, local administrative units, were randomly selected from a total of 38 using a lottery method. Next, children were proportionally allocated to each selected kebele based on their population size. Finally, systematic sampling was conducted within each kebele. A sampling interval (K) was determined by dividing the total number of children (N) by the allocated sample size (n). The first participant was randomly chosen, and subsequent participants were selected at intervals of K.

### Operational and term definitions

**Minimum Dietary Diversity for children aged 6–23 months (MDD-C):** This dichotomous indicator was developed by the WHO and UNICEF to be used as a proxy indicator to ensure that nutrient needs are met among children aged 6–23 months. It is assessed using eight food groups, which include breast milk; grains, roots, tubers, and plantains; pulses (beans, peas, and lentils); nuts and seeds; dairy products (milk, infant formula, yogurt, and cheese); flesh foods (meat, fish, poultry, and organ meats); eggs; vitamin-A-rich fruits and vegetables; and other fruits and vegetables. The variety of food groups consumed ranges from at least one to a maximum of eight. If a child consumed at least five of the eight defined food groups within the preceding 24 hours, the child was considered to have adequate MDD; otherwise, it was considered to have inadequate MDD [12].

**Recommended Dietary Allowances (RDA):** This is the recommended daily nutrient consumption that meets the nutritional needs of nearly all (97.5%) children [26].

**Nutrient Adequacy Ratio (NAR):** The NAR is the ratio of a subject’s micronutrient consumption to the current RDA for each sex and age group [13].

**Mean Adequacy Ratio (MAR):** The MAR is a comprehensive metric that serves as an indicator of overall diet quality. It was calculated by dividing the sum of all NAR values by the total count of computed micronutrients [13].

**Micronutrient intake inadequacy:** The incidence occurred when children consumed less than 100% of the RDA for a certain micronutrient, and the NAR for that nutrient was less than one [13].

**Overall micronutrient intake inadequacy:** The ideal MAR cutoff for nutrient intake inadequacy should be one (100%), which would mean that the intake of all 12 nutrients, namely, vitamin A, vitamin B1, vitamin B2, vitamin B3, vitamin B6, vitamin B9, vitamin B12, vitamin C, calcium, iron, zinc, and selenium, is equal to or greater than the RDA and that the requirements for all the nutrients are met. In this study, since there were no participants who had a MAR score of 1, overall micronutrient intake inadequacy was operationalized to be < 0.75 [13,27–29].

### Data collection tools and procedures

The data were collected using an interviewer-administered structured questionnaire via the Kobo Toolbox, which is an electronic data collection toolkit. A group of 10 skilled data collectors, assisted by two experienced supervisors with expertise in public health nutrition, actively participated in the data collection and supervision processes.

#### 24-hour dietary recall assessment.

Before collecting actual data, home surveillance and market inspections were carried out to determine the types of foods consumed, cooking processes, and kitchen tools used in the study area. During the surveillance, photographs of household utensils and food portions consumed during a single meal were taken, and each item received a code. In the nutrition laboratory, devices for serving food were standardized with a digital food portion weighing scale and a measuring cylinder containing food portions and water [29].

During data collection, respondents were asked about the utensils they used and estimated food portions while referring to a photographic atlas. The atlas contained photos of various household utensils, such as spoons, ladles, cups, glasses, and bowls, as well as different food portions. These photos helped participants remember and identify the types and sizes of the food they ate. An interactive single multiple-pass 24-hour recall approach was used. In addition, quantities of consumed food items, such as oranges, bananas, mangos, and potatoes, that could be counted were determined using numbers. These foods were then classified into large, medium, and small categories based on their size [29]. The food consumption data were then transformed into nutrient intake data using food composition tables, which provided estimates of nutrient content per 100 grams of each food item [30–33].

### Data quality control

To ensure consistency, the questionnaire was prepared in English first, then translated into Amharic, and then back into English. A pretest was administered to approximately 5% of the sample, drawn from a population outside the actual study sample. Supervisors and data collectors received training, and the data collection process was rigorously supervised to ensure data quality.

Multiple-pass, 24-hour recall was used to collect dietary data. This technique consisted of three steps: first, a “quick list” was made; second, a thorough description of all food and beverage items consumed was documented; and third, a review was performed.

### Data processing and analysis

Following data collection, the food consumption data were transformed into nutrient intake data using the NutriSurvey 2007 software [34]. Ethiopian food composition tables were used to estimate the nutrient content per 100 grams of each food item [30,31]. When certain food items were not included in the Ethiopian food composition tables, alternative tables from other African countries, such as Kenya [32] and Tanzania [33], were used as a reference. NutriSurvey 2007 software was used to analyze the nutrient intake data, and the remaining analysis was conducted using SPSS version 25.

The RDA, established by the WHO and the Food and Agriculture Organization (FAO) in 2004, was utilized to compare and evaluate actual nutrient intake. The NAR was then calculated to determine the adequacy of a specific nutrient. In addition, the MAR was used to evaluate overall micronutrient intake adequacy. The MAR considers 12 micronutrients selected for their importance to public health, including vitamin A, thiamin, riboflavin, niacin, vitamin B-6, folate, vitamin B-12, vitamin C, calcium, iron, zinc, and selenium [13,29,35].


$$\text{NAR}=\frac{\text{Actual intake of the nutrient per day }}{\text{RDA of that nutrient}}$$

To determine the overall adequacy of micronutrients, the MAR was computed as follows:


$$\text{MAR}=\frac{\sum \text{NAR }(\text{each truncated at }\text{1})}{\text{Number of nutrients}}$$

The NAR was capped at one, indicating that a nutrient with a higher NAR was unable to compensate for another nutrient with a lower NAR. To assess the adequacy of individual nutrients, NAR was calculated, with adequacy defined as meeting or exceeding the RDA. Overall micronutrient adequacy was then determined using the MAR, calculated as the ratio of the sum of all NARs to the total number of nutrients assessed. A MAR value equal to one indicates adequate micronutrient intake for a child [13,29]. Kolmogorov‒Smirnov and Shapiro‒Wilk tests were used to assess the normality of micronutrient intake data (p-value >0.05). The data were not normally distributed; hence, the results were presented as the median and interquartile range.

The validity of the MDD-C was evaluated by comparison with that of the MAR, the gold standard for high accuracy. The choice of statistical methods was aligned with the study’s aim to assess the indicator’s criterion validity and its ability to predict overall micronutrient adequacy. Because the MAR distribution was skewed, Spearman’s rank correlation test was used to determine the correlation between MDD and the MAR. The strength of the correlation was interpreted using the correlation coefficient as follows: poor (0–0.29), fair (0.30–0.59), moderate (0.60–0.79), and strong (0.80–1.00) correlation [36,37]. To assess the level of agreement between the MDD-C and the adequacy of micronutrients, Cohen’s kappa was used. The kappa scores were interpreted as follows: poor agreement (<0.00), slight agreement (0.00–0.20), fair agreement (0.21–0.40), moderate agreement (0.41–0.60), substantial agreement (0.61–0.80), and almost perfect agreement (0.81–1.00) [38].

In addition, sensitivity and specificity tests were performed to determine the accuracy of the MDD-C in detecting children with high MAR values. The Area Under the Curve (AUC) was computed using the Receiver Operating Characteristic (ROC) curve based on nutrient adequacy as yes (MAR ≥ 0.75) or no (MAR < 0.75). The AUC values were then interpreted according to predefined criteria, which categorized them as fail (0.5–0.6), poor (0.6–0.7), fair (0.7–0.8), good (0.8–0.9), or excellent accuracy (0.9–1.0) [39]. The optimal cutoff points were identified by selecting the points that maximized the Youden J statistic (sensitivity + specificity – 1) (the larger, the better) [40]. All assumptions for the applied statistical tests were checked and met. A p-value < 0.05 was considered to indicate statistical significance. The final results are presented in text, tables, and graphs.

### Ethical considerations

The study was conducted following the Declaration of Helsinki and was approved by the Ethical Review Board of the College of Medicine and Health Science at Bahir Dar University (protocol Code: 631/2023 and date of approval: February 2, 2023). The Amhara Public Health Institute offered a permission letter, and the North Mecha District administration office provided a formal letter of cooperation to the selected kebeles. Before collecting the data, the mothers of the children were asked to provide informed written consent. The confidentiality of the data and the privacy of the study participants were upheld.

## Results

### Sociodemographic and economic characteristics

A total of 430 children aged 6–23 months participated in the study, with a 94.1% response rate. The mean (± SD) age of children and mothers was 10.9 (±3.5) months and 29.46 (±5.55) years, respectively. A little above half (51.2%) were females. All (100%) of the study participants were followers of the Orthodox Christian religion. The mean (± SD) family size, maternal parity, and number of children were 5.8 (± 1.9), 3.9 (± 2.0), and 1.3 (± 0.5), respectively (Table 1).

**Table 1:** Sociodemographic characteristics of children aged 6–23 months in North Mecha District, Northwest Ethiopia, 2023 (N = 430).

Variables	Categories	Frequency	Percentage
Child age in months (Mean ± SD: 10.9 ± 3.5)	6-8	137	31.9
	9-12	152	35.3
	13-23	141	32.8
Child sex	Male	210	48.8
	Female	220	51.2
Maternal age in years (Mean ± SD: 29.5 ± 5.6)	18-25	119	27.7
	26-35	258	60.0
	36-50	53	12.3
Maternal education	Unable to read and write	319	74.2
	Primary school incomplete	40	9.3
	Primary school completed	42	9.8
	Secondary school completed	24	5.6
	University or college completed	5	1.2
Maternal occupation	Farmer	333	77.4
	Merchant	17	4.0
	Housewife	78	18.1
	Employee	2	0.5
Maternal marital status	Married	423	98.4
	Widowed	7	1.6
Paternal education (N = 423)	Unable to read and write	193	44.9
	Primary school incomplete	165	38.4
	Primary school completed	44	10.2
	Secondary school completed	17	4.0
	University or college completed	4	0.9
Paternal occupation (N = 423)	Farmer	390	90.7
	Merchant	15	3.5
	Student	4	0.9
	Daily laborer	7	1.6
	Other^#^	7	1.6
Family size (Mean ± SD: 5.8 ± 1.9)	≤4	123	28.6
	5-7	224	52.1
	≥8	83	19.3
Parity (Mean ± SD: 3.9 ± 2.0)	≤2	123	28.6
	3-5	209	48.6
	≥6	98	22.8
Number of children <5 (Mean ± SD: 1.3 ± 0.5)	1	300	69.8
	≥2	130	30.2

*SD: Standard Deviation;* ^*#*^*Driver, Soldier, Veterinarian.*

### Minimum dietary diversity and nutrient adequacy of children

**Fig 1:**
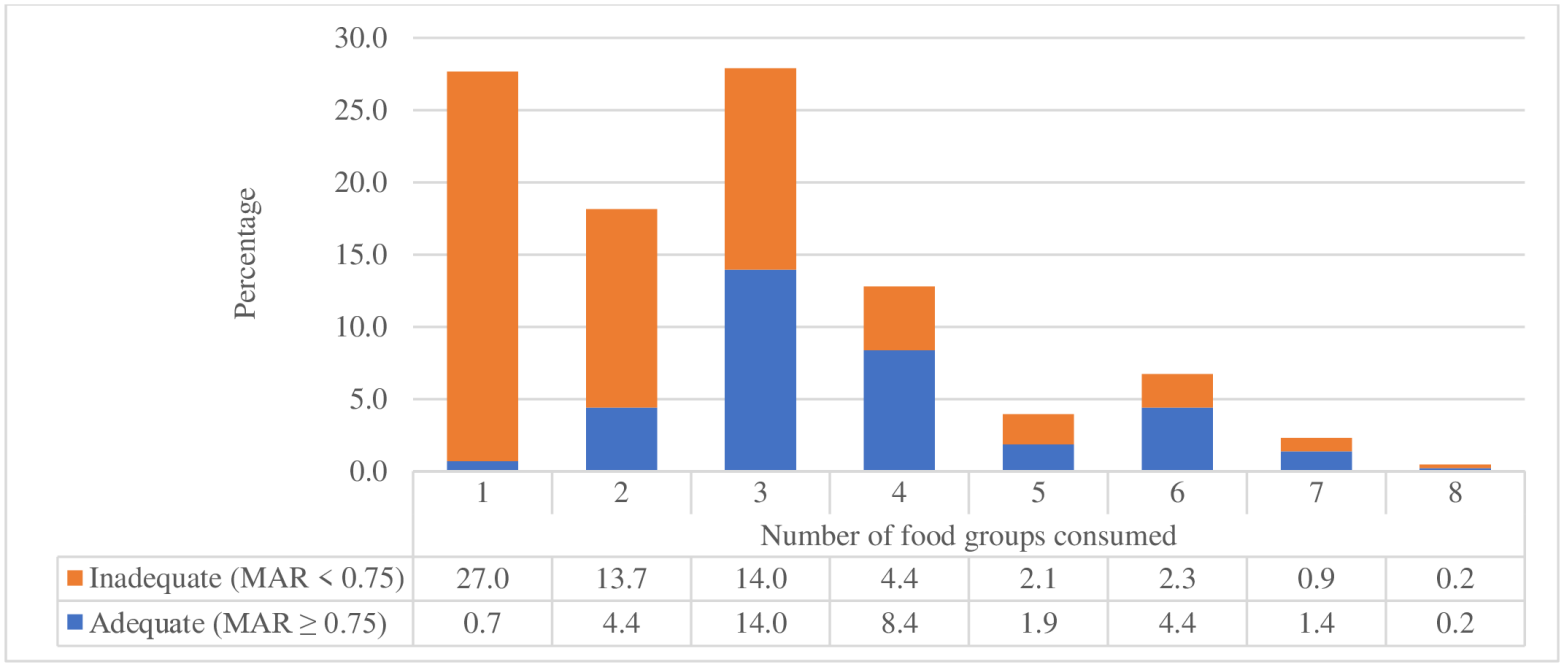
Number of consumed food groups among children aged 6-23 months in North Mecha District, Northwest Ethiopia, 2023 (N = 430).

**Fig 2:**
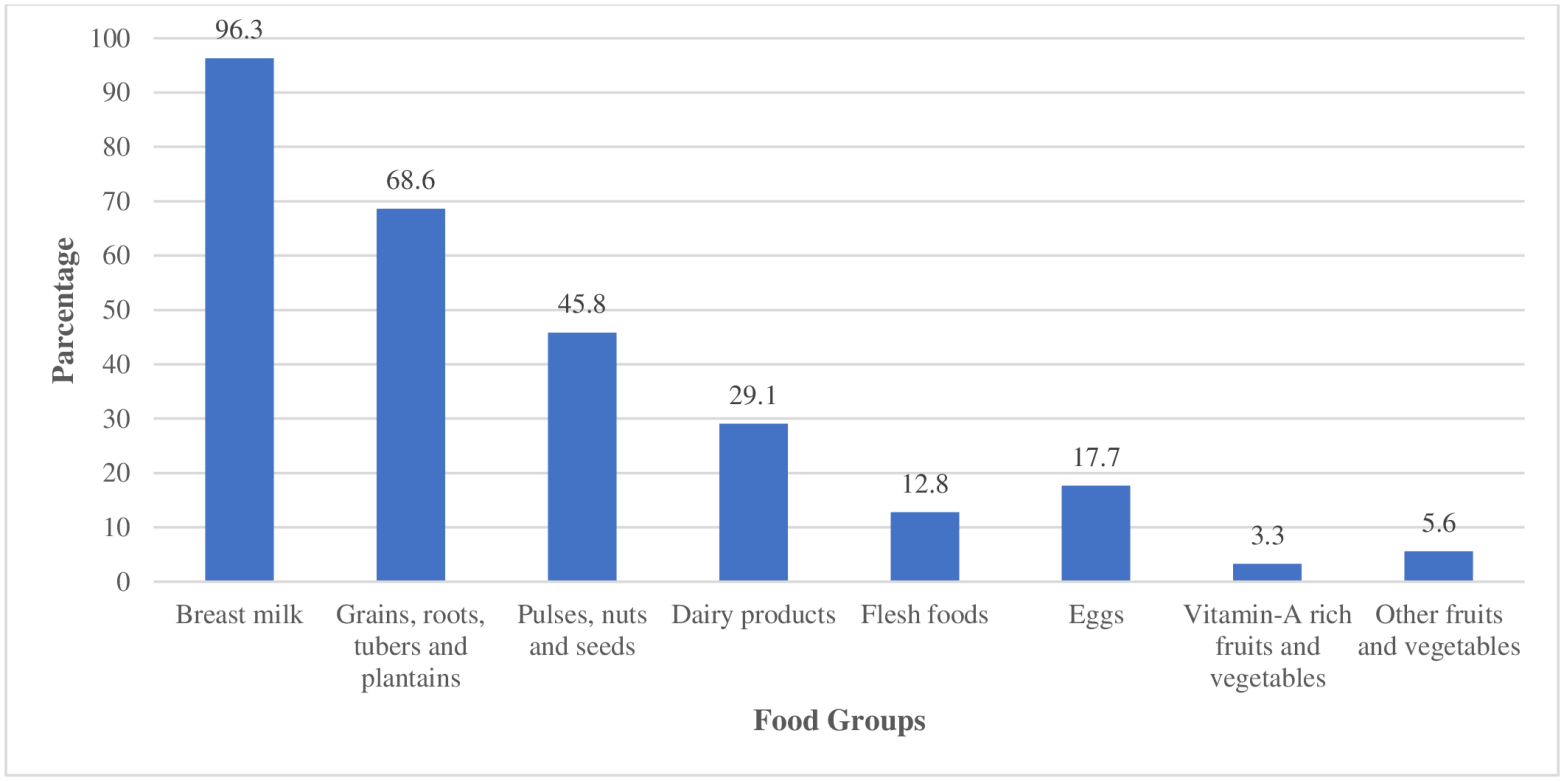
Types of consumed food groups among children aged 6-23 months in North Mecha District, Northwest Ethiopia, 2023 (N = 430).

The variety of food groups consumed ranges from at least one to a maximum of eight. The prevalence of having MDD (≥5 food groups) was 13.5% (95% CI: 10.4, 17.1) in the 24 hours before the survey. The median (± IQR) MDD score was 3.0 (± 3.0) (Fig 1). Most (96.3%) of the study participants consumed breast milk, while the lowest percentage (3.3%) consumed vitamin-A-rich fruits and vegetables (Fig 2). Only one child (0.2%) met the recommended levels of all 12 nutrients (MAR of one). The overall prevalence of micronutrient intake adequacy, MAR ≥ 0.75, was 35.3% (95% CI: 30.8, 40.1) (Fig 1).

### Validation of the performance of the MDD-C to predict micronutrient intake adequacy

**Fig 3:**
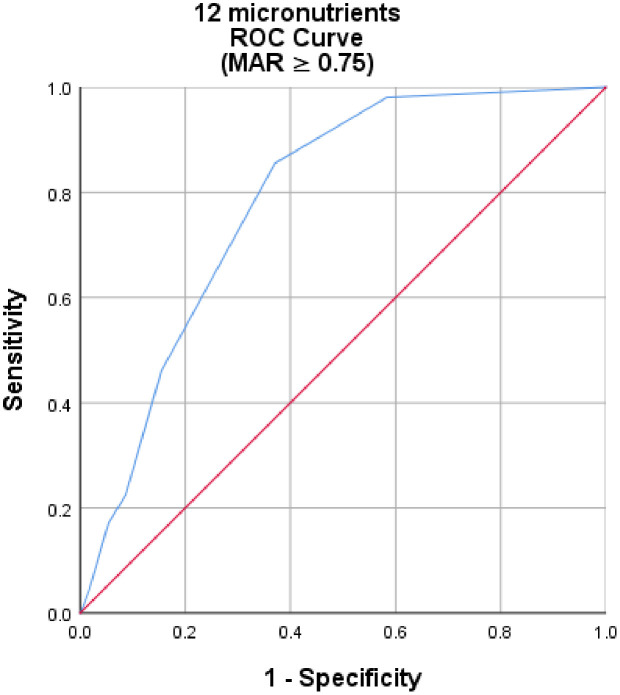
The ROC curve illustrates the performance of dietary diversity for children in predicting adequate micronutrient intake among children aged 6-23 months in North Mecha District, Northwest Ethiopia, 2023 (MAR ≥ 0.75) (N = 430).

The performance of the MDD-C was evaluated at various MAR thresholds. The AUC ranged from 0.79 to 0.83, indicating either fair or good accuracy. Spearman correlation analysis revealed a statistically significant, moderate positive correlation between the MDD and the MAR when determining micronutrient intake adequacy (ρ = 0.62, p < 0.001). ROC curve analyses indicated that MDD had a fair predictive ability for micronutrient adequacy concerning the overall adequacy of 12 micronutrients at a MAR ≥ 0.75 (AUC = 0.78, 95% CI: 0.74, 0.83) (p < 0.001) (Fig 3; Table 2).

**Table 2:** Summary of dietary diversity for children indicator characteristics at various MAR thresholds among children aged 6-23 months in North Mecha District, Northwest Ethiopia, 2023 (N = 430).

Threshold	*ρ*	AUC	MDD optimal cutoff (≥)	Value at the optimal cutoff			
				Sensitivity (%)	Specificity (%)	J	Kappa
MAR ≥ 0.50	0.44***	0.80 (0.75, 0.86)***	2	84.9	73.1	0.58	0.527***
MAR ≥ 0.65	0.55***	0.81 (0.77, 0.85)***	2	97.3	54.6	0.52	0.527***
MAR ≥ 0.67	0.54***	0.80 (0.76, 0.85)***	2	97.3	53.8	0.51	0.516***
MAR ≥ 0.75	0.48***	0.78 (0.74, 0.83)***	3	85.5	62.9	0.49	0.433***
MAR ≥ 0.85	0.41***	0.79 (0.74, 0.83)***	3	93.3	56.0	0.49	0.308***
MAR ≥ 0.95	0.25***	0.83 (0.77, 0.90)***	4	85.7	76.8	0.63	0.203***

*ρ: Spearman rank correlation coefficient (rho); AUC: Area Under the Curve; J: Youden index; MDD: Minimum Dietary Diversity*.

*P value <0.05; **P value <0.01; ***P value <0.001.

### Determination of the optimal cutoff for dietary diversity

The optimal cutoff increased from two to four as the MAR threshold increased from 0.50 to 0.95, but the level of agreement decreased from moderate to fair. The best balance between sensitivity and specificity was found when the MAR was ≥ 0.95 at the optimal cutoff of four for MDD-C (Table 2).

The MDD-C score (≥5 food groups), which is a proxy indicator developed by the WHO and UNICEF to reflect micronutrient adequacy, had a sensitivity of 22.4% and a specificity of 91.4%. Based on the findings of this study, as determined by considering the Youden index, the optimal cutoff point for MDD-C to predict micronutrient intake adequacy was ≥ 3 food groups at a MAR ≥ 0.75. This resulted in a sensitivity of 85.5% and a specificity of 62.9%. The Kappa statistics also showed that there was a relatively greater Kappa value when examining the dietary diversity of ≥3 food groups than when examining the dietary diversity of all other cutoff groups, including the recommended ≥5 food groups. This suggests moderate agreement between consuming ≥3 food groups and meeting essential micronutrient requirements (Table 3).

**Table 3:** Summary of dietary diversity for children indicator characteristics for 10 food groups among children aged 6-23 months in North Mecha District, Northwest Ethiopia, 2023 (MAR ≥ 0.75) (N = 430).

Food Groups	Sensitivity (%)	Specificity (%)	PPV (%)	NPV (%)	J	Kappa
≥1	100	0	35.3	0	0	0.000
≥2	98.0	41.7	47.9	97.5	0.40	0.321***
≥3	85.5	62.9	55.8	88.8	0.49	0.433***
≥4	46.1	84.5	61.9	74.1	0.31	0.325***
≥5	22.4	91.4	58.6	68.3	0.14	0.160***
≥6	17.1	94.6	63.4	67.6	0.12	0.140***
≥7	4.6	98.2	58.3	65.3	0.03	0.035
≥8	0.7	99.6	50.0	64.7	<0.01	0.004

*PPV: Predictive Value Positive; NPV: Negative Predictive Value; J: Youden Index.*

*p value <0.05; **p-value <0.01; ***p value <0.001.

Spearman rank correlation analysis revealed that dietary diversity in children had a significant moderate positive correlation with vitamin B3, iron, and selenium. Among the other nutrients that showed significant positive correlations, dietary diversity had poor (vitamin B9) and fair (vitamins B1, B2, and B6, calcium, and zinc) correlations. ROC analyses revealed that the AUCs for all micronutrients with significant values and positive correlations ranged from 0.64 to 0.78, with the highest score observed for vitamin B2 (S1–S12 Figs). Dietary diversity had a fair predictive ability to predict nutrient intake adequacy for all micronutrients, with a significant positive correlation, except for calcium, which had poor predictive ability. The Youden index revealed that, similar to the overall optimal cutoff for MDD-C to predict micronutrient intake adequacy, the optimal cutoff point for MDD was ≥ 3 food groups for all micronutrients with a positive correlation and significant predictive ability, except for calcium, which had an optimal cutoff of ≥2 food groups (Table 4).

**Table 4:** Summary of the dietary diversity for children indicator characteristics for each micronutrient among children aged 6-23 months in North Mecha District, Northwest Ethiopia, 2023 (NAR = 1) (N = 430).

Micronutrient	*ρ*	AUC	MDD optimal cutoff (≥)	Value at the optimal cutoff			
				Sensitivity (%)	Specificity (%)	J	Kappa
Vitamin A	−0.07	0.45 (0.39, 0.52)	6	10.2	92.7	0.03	0.014
Vitamin B1	0.47***	0.74 (0.69, 0.80)***	3	85.5	51.8	0.37	0.190***
Vitamin B2	0.41***	0.78 (0.73, 0.82)***	3	86.0	56.3	0.42	0.304***
Vitamin B3	0.64***	0.71 (0.65, 0.77)***	3	83.3	50.5	0.34	0.153***
Vitamin B6	0.47***	0.73 (0.69, 0.78)***	3	79.9	58.7	0.39	0.335***
Vitamin B9	0.19***	0.74 (0.68, 0.79)***	3	88.9	51.8	0.41	0.192***
Vitamin B12	−0.13**	0.41 (0.35, 0.47)**	7	3.3	98.4	0.02	0.009
Vitamin C	−0.05	0.44 (0.35, 0.53)	6	10.0	96.7	0.07	0.010
Calcium	0.35***	0.64 (0.57, 0.71)**	2	95.7	30.5	0.26	0.076***
Iron	0.64***	0.77 (0.73, 0.82)***	3	83.0	65.7	0.49	0.464***
Zinc	0.56***	0.71 (0.64, 0.78)***	3	85.3	48.5	0.34	0.092***
Selenium	0.66***	0.77 (0.73, 0.81)***	3	82.0	63.5	0.46	0.425***
12 Nutrients	0.62***	0.78 (0.74, 0.83)***	3	85.5	62.9	0.49	0.433***

*ρ: Spearman rank correlation coefficient (rho); AUC: Area Under the Curve; J: Youden Index; MDD: Minimum Dietary Diversity.*

*P value <0.05; **P value <0.01; ***P value <0.001.

## Discussion

Adequate intake of micronutrients by children aged 6–23 months determines their nutritional status and ensures the needed growth and development, as well as future health and productivity [1,2]. It is crucial to validate the effectiveness of easily measurable indicators used to reflect the adequacy of micronutrients. Limited evidence on the effectiveness of the MDD indicator in predicting micronutrient adequacy among infants and young children has highlighted the need for validation of the MDD indicator, including in Ethiopia, a country with varied dietary consumption habits. Therefore, this study aimed to validate the performance of MDD to predict micronutrient adequacy against the MAR among children aged 6–23 months in the North Mecha District, Northwest Ethiopia.

This study revealed that the MDD-C had a statistically significant moderate positive correlation and a fair ability to predict the overall adequacy of intake of 12 micronutrients (MAR ≥ 0.75). This suggests that MDD-C can serve as a practical and reasonably accurate proxy for monitoring dietary adequacy in resource-limited settings. Similarly, a study in the Philippines showed a fair positive correlation between dietary diversity and Mean Probability Adequacy (MPA) [22]. Furthermore, a study conducted in rural Benin showed that dietary diversity predicted the mean adequacy of micronutrient density, regardless of season [21]. A systematic review, including studies conducted in low-income settings across African and Asian countries, where dietary diversity is often limited, validated the performance of the 7-food-group dietary diversity indicator prior to the development of the 2021 IYCF indicators. The review concluded that the dietary diversity of infants and children under 5 years old was positively associated with micronutrient adequacy [19]. Overall, although the strength of association may vary, MDD-C demonstrates strong potential as a practical and reliable tool for monitoring and improving child nutrition through enhanced dietary diversity, particularly in public health programs operating in resource-limited settings where detailed dietary assessments are not feasible. Dietary diversity increases the likelihood of meeting micronutrient requirements and helps prevent deficiencies, as various food groups provide complementary nutrients essential for optimal growth and development in young children. A diversified diet enhances nutrient absorption, supports immune and cognitive development, and reduces the risk of hidden hunger during the critical growth window of 6–23 months. Evidence shows that low dietary diversity, particularly limited intake of nutrient-dense foods such as eggs, dairy, fruits, and vegetables, is associated with micronutrient deficiencies, stunting, and impaired physical and cognitive outcomes in young children [12,41–43]. Thus, dietary diversity is not only a proxy indicator but also a vital component of optimal child nutrition.

The MDD-C score (≥5 food groups), which is a proxy indicator developed by the WHO and UNICEF to reflect micronutrient adequacy, had a sensitivity of 22.4% and a specificity of 91.4% [12]. This finding implies that the MDD-C is a very precise tool for evaluating the adequacy of micronutrients at a defined threshold. This suggests that using the MDD-C indicator with a cutoff of ≥5 food groups is more reliable for ensuring that individuals who meet the criteria for MDD-C and test positive are likely to have adequate intake of micronutrients based on the MAR score. Using this cutoff reduces the likelihood of false positive results, in which infants and young children are incorrectly classified as having adequate micronutrient intake when they have a low probability of it (<5 food groups). However, this threshold has disadvantages due to its low sensitivity. This indicates that it may not accurately identify and may miss children with adequate micronutrient intake levels, potentially resulting in misdiagnosis and failure to recognize those who meet the specified MDD-C criteria. This has significant implications for child nutrition, potentially undermining targeted nutritional support and program recognition while reducing the effectiveness of nutritional interventions at both individual and population levels. Moreover, it directly or indirectly impacts long-term health outcomes, as misdiagnoses can hinder efforts to promote optimal growth, cognitive development, and overall well-being.

The sensitivity of the MDD-C indicator (≥5 food groups) was significantly lower in the current study than that of a similar indicator calculated from 8 food groups with MPA ≥ 1, as indicated in a study conducted in the Philippines. Conversely, the specificity of the MDD-C indicator (≥5 food groups) in the present study was greater than that reported in a similar study in the Philippines [22]. Despite both studies having used a single 24-hour recall, the difference could have been attributed to variations in factors such as dietary patterns, socioeconomic and cultural characteristics, and the thresholds used to define adequacy.

Among the individual nutrients that showed significant positive correlations, dietary diversity in children had a significant, moderate positive correlation with vitamin B3, iron, and selenium. Among other nutrients, dietary diversity had poor correlations with vitamin B9 and fair correlations with vitamins B1, B2, and B6, calcium, and zinc. Dietary diversity had fair accuracy in predicting nutrient intake adequacy for all micronutrients with significant AUC values and positive correlations, except for calcium, which had poor accuracy. This suggests that relying on the variety of eight food groups in the diet is not an accurate method for predicting the adequacy of vitamins A, B12, and C. Therefore, it implies that MDD-C may not be equally representative of all micronutrients for which it was originally developed.

The optimal cutoff point for MDD-C to predict micronutrient intake adequacy based on the findings of the present study at a MAR ≥ 0.75, determined by considering the Youden index, was ≥ 3 food groups, resulting in a sensitivity of 85.5% and a specificity of 62.9%. The Kappa statistics also demonstrated that a relatively higher Kappa value was observed when examining the dietary diversity of ≥3 food groups. This finding showed moderate agreement between the consumption of ≥3 food groups and meeting essential micronutrient requirements. The Youden index also revealed that, similar to the overall optimal cutoff for MDD to predict micronutrient intake adequacy, the optimal cutoff point for MDD-C was ≥ 3 food groups for all micronutrients with positive correlation and significant predictive ability (vitamins B1, B2, B3, B6, and B9; iron; zinc; and selenium), except for calcium, which had an optimal cutoff of ≥2 food groups.

In accordance with the moderate agreement observed between the MDD-C (≥3 food groups) and the overall adequacy of micronutrients using the MAR, the MDD-C (≥3 food groups) for individual micronutrients also showed moderate agreement with the adequacy of iron and selenium. The other nutrients with significant agreement showed fair (vitamin B2 and B6) to slight agreement (vitamin B1, B3, B9, calcium, and zinc) at the same cutoff (≥3 food groups), except for calcium, which showed slight agreement with an optimal cutoff of ≥2 food groups. In general, this study showed that the MDD-C (≥3 food groups) indicator had better sensitivity than the MDD-C (≥5 food groups) indicator, which had high specificity but low sensitivity. Ultimately, the MDD-C (≥3 food groups) indicator was found to have a better balance between sensitivity and specificity, making it the preferred cutoff point.

Supporting the lower optimal cutoff identified in our study, previous validation studies in Ethiopia, Burkina Faso, and Benin, which assessed the performance of earlier dietary diversity indicators before the development of the new MDD-C for children, reported optimal thresholds below the standard recommendation [20,21,44]. Furthermore, although evidence on the performance of the new MDD-C in Ethiopia is limited, recent studies among women have also similarly proposed cutoffs below the recommended ≥5 food groups for achieving MDD [45,46]. These findings reinforce the contextual relevance of adopting a lower cutoff in settings with low dietary diversity. However, the optimal cutoff determined in this study is lower than the optimal cutoff indicated in a study conducted in the Philippines [22]. The study showed that the MDD-C score of five was the cutoff that maximized sensitivity and specificity in accurately identifying breastfed children with 100% MPA. The first possible reason for the variation observed could be related to the threshold of the MAR chosen to determine the level of adequacy required to be considered sufficient.

When evaluating the MDD-C indicator with a uniform cutoff, the sensitivity tends to improve with increasing MAR thresholds, while the specificity tends to decrease. Similarly, increasing the cut-off of MDD increased sensitivity at the expense of specificity and increased the percentage of misclassification. These determinations had little impact on finding the optimal balance between sensitivity and specificity and establishing the most suitable cutoff point. Nutrient adequacy depends not only on dietary diversity but also on the portion size of monotonous foods and the frequency of their consumption. Thus, a lower optimal cutoff may better reflect the MDD-C in such contexts. Overall, factors such as cultural differences in dietary practices, methodological discrepancies, and socioeconomic influences all significantly contributed to the observed variations in the study [12,22,45,46]. However, in this study, RDA was used instead of Estimated Average Requirement (EAR), which may result in a higher expected intake to meet the recommended dietary reference intake. This could compensate for the difference resulting from the MAR threshold variation. In contrast, it is worth noting that this anticipation contrasts with the findings of a previous study [22].

The discrepancies observed in studies and divergences from the recommendations of the WHO and UNICEF on IYCF indicators related to the MDD-C cutoff for defining micronutrient adequacy have significant implications. These include challenges in accurately identifying individuals with sufficient micronutrient intake, making informed resource allocation decisions, effectively monitoring and evaluating programs, and shaping evidence-based policy. Consequently, as countries integrate the IYCF guideline into their healthcare systems, it is estimated that discrepancies in this aspect will arise and require careful consideration.

Overall, the finding that the revised MDD-C serves as a fair predictor of micronutrient adequacy among Ethiopian children aged 6–23 months carries important implications for both global and national nutrition agendas. Given its recent inclusion as a Sustainable Development Goal (SDG) indicator, MDD-C facilitates international comparisons while also offering a practical measure for national policy formulation, program design, and community-based interventions [47]. Although its performance at the standard WHO/UNICEF cutoff was suboptimal, this study underscores the potential for improving its predictive utility through context-specific adaptations of the cutoff point [12].

Importantly, the continued use of MDD-C in routine nutrition surveillance and program monitoring is justified, particularly in low-resource settings where comprehensive dietary assessments are often not feasible. Due to its simplicity, affordability, and ease of integration, MDD-C can be embedded within existing community-level platforms—such as growth monitoring and behavior change communication strategies—as a tool to identify children at risk of micronutrient inadequacy and to assess the impact of interventions promoting dietary diversity. While the global cutoff provides a standardized benchmark, the findings from this study highlight the necessity for further research to establish and validate context-specific thresholds, thereby enhancing the accuracy of dietary adequacy assessments across diverse populations [12].

### Strengths and limitations

This study validated the performance of MDD-C, endorsed by the WHO and UNICEF [12], to predict the adequacy of micronutrient intake. It focuses on children aged 6–23 months in Ethiopia, as their consumption affects their nutritional status and overall health status in the present and future. The results of the present study contribute to addressing the existing evidence gap by validating the predictive value of MDD-C for micronutrient adequacy among Ethiopian children. However, the reliance on a single 24-hour recall may introduce potential inaccuracies due to variations in daily eating patterns, seasonal variations, and memory biases. To mitigate this issue, rigorous efforts have been implemented to integrate standardized quality-control measures throughout the entirety of the research procedure. Skilled nutrition professionals have participated to guarantee precision and uniformity in the data collection process. Moreover, special event days were excluded from the data collection process to ensure that the conclusions drawn accurately represented the usual dietary pattern among the subjects.

## Conclusions

The MDD-C had a statistically significant, moderate positive correlation and fair predictive ability with the MAR in accurately predicting the adequacy of micronutrient intake at the optimal cutoff. The optimal cutoff point for MDD-C to predict adequate micronutrient intake was found to be ≥ 3 food groups. At the standard cutoff of ≥5 food groups, MDD-C exhibited high specificity but poor sensitivity, indicating limited ability to correctly identify children with adequate micronutrient intake. This deviation of the optimal cutoff point from the recommended cutoff point of the MDD-C in guidelines for assessing IYCF practices and predicting micronutrient adequacy has significant implications for its application. Countries need to adapt MDD-C cutoffs to local dietary patterns to improve accuracy. As the lead agencies for this indicator, WHO and UNICEF should revisit the global application of a uniform cutoff and support the development of context-specific thresholds. Such adaptations would strengthen the indicator’s utility across diverse populations and enhance its role in informing nutrition policies and programs, particularly in low- and middle-income countries. In addition to the relevant findings of this study in the northwestern part of Ethiopia, further studies are needed to reassess the effectiveness of MDD-C as a proxy indicator for determining micronutrient adequacy in diverse populations across multiple countries.

## Supporting information

S1 Fig The ROC curve illustrates the performance of dietary diversity for children in predicting adequate micronutrient intake of vitamin A among children aged 6–23 months in North Mecha District, Northwest Ethiopia, 2023 (NAR = 1) (N = 430). (TIF)

S2 Fig The ROC curve illustrates the performance of dietary diversity for children in predicting adequate micronutrient intake of vitamin B1 among children aged 6–23 months in North Mecha District, Northwest Ethiopia, 2023 (NAR = 1) (N = 430). (TIF)

S3 Fig The ROC curve illustrates the performance of dietary diversity for children in predicting adequate micronutrient intake of vitamin B2 among children aged 6–23 months in North Mecha District, Northwest Ethiopia, 2023 (NAR = 1) (N = 430). (TIF)

S4 Fig The ROC curve illustrates the performance of dietary diversity for children in predicting adequate micronutrient intake of vitamin B3 among children aged 6–23 months in North Mecha District, Northwest Ethiopia, 2023 (NAR = 1) (N = 430). (TIF)

S5 Fig The ROC curve illustrates the performance of dietary diversity for children in predicting adequate micronutrient intake of vitamin B6 among children aged 6–23 months in North Mecha District, Northwest Ethiopia, 2023 (NAR = 1) (N = 430). (TIF)

S6 Fig The ROC curve illustrates the performance of dietary diversity for children in predicting adequate micronutrient intake of vitamin B9 among children aged 6–23 months in North Mecha District, Northwest Ethiopia, 2023 (NAR = 1) (N = 430). (TIF)

S7 Fig The ROC curve illustrates the performance of dietary diversity for children in predicting adequate micronutrient intake of vitamin B12 among children aged 6–23 months in North Mecha District, Northwest Ethiopia, 2023 (NAR = 1) (N = 430). (TIF)

S8 Fig The ROC curve illustrates the performance of dietary diversity for children in predicting adequate micronutrient intake of vitamin C among children aged 6–23 months in North Mecha District, Northwest Ethiopia, 2023 (NAR = 1) (N = 430). (TIF)

S9 Fig The ROC curve illustrates the performance of dietary diversity for children in predicting adequate micronutrient intake of calcium among children aged 6–23 months in North Mecha District, Northwest Ethiopia, 2023 (NAR = 1) (N = 430). (TIF)

S10 Fig The ROC curve illustrates the performance of dietary diversity for children in predicting adequate micronutrient intake of iron among children aged 6–23 months in North Mecha District, Northwest Ethiopia, 2023 (NAR = 1) (N = 430). (TIF)

S11 Fig The ROC curve illustrates the performance of dietary diversity for children in predicting adequate micronutrient intake of zinc among children aged 6–23 months in North Mecha District, Northwest Ethiopia, 2023 (NAR = 1) (N = 430). (TIF)

S12 Fig The ROC curve illustrates the performance of dietary diversity for children in predicting adequate micronutrient intake of selenium among children aged 6–23 months in North Mecha District, Northwest Ethiopia, 2023 (NAR = 1) (N = 430). (TIF)

## Abbreviations

AUC: Area Under the Curve
FAO: Food and Agriculture Organization
IQR: Interquartile Range
IYCF: Infant and Young Child Feeding
MAR: Mean Adequacy Ratio
MDD: Minimum Dietary Diversity
MDD-C: Minimum Dietary Diversity for Children aged 6-23 months
NAR: Nutrient Adequacy Ratio
NPV: Negative Predictive Value
PPV: Predictive Value Positive
RDA: Recommended Daily Allowance
ROC: Receiver Operating Characteristic
Sn: Sensitivity
UNICEF: United Nations Children’s Fund
WHO: World Health Organization.
