# Supplementary figures and images for: Minimum Dietary Diversity for Children aged 6–23 months as a predictor of micronutrient adequacy in Ethiopia: Validation of the proxy indicator
Source: PLoS One. 2025 Oct 17;20(10):e0334827. doi: 10.1371/journal.pone.0334827 (PMC12533918; doi:10.1371/journal.pone.0334827)

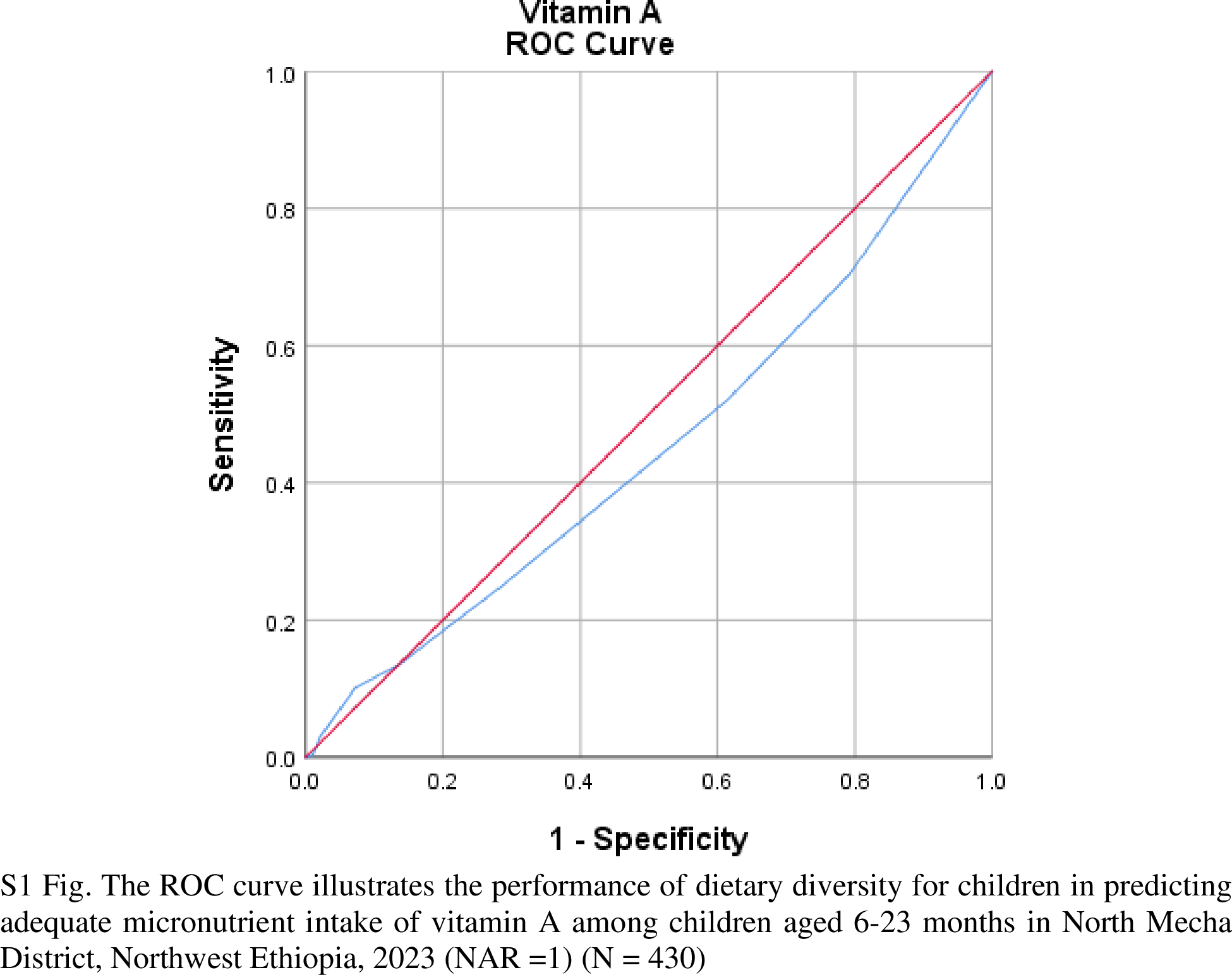

Supplement: S1 Fig — (TIF) [file pone.0334827.s001.tif]

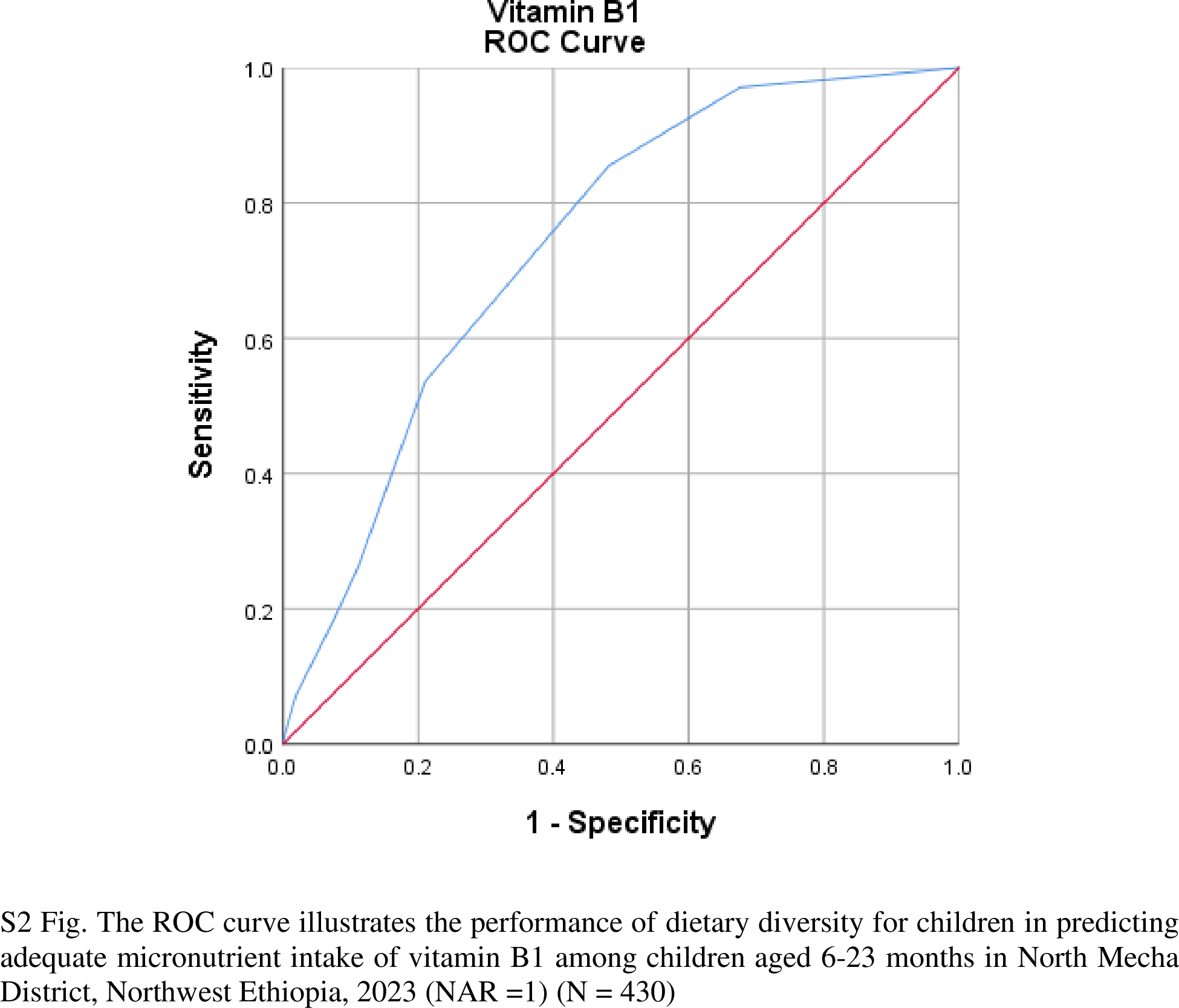

Supplement: S2 Fig — (TIF) [file pone.0334827.s002.tif]

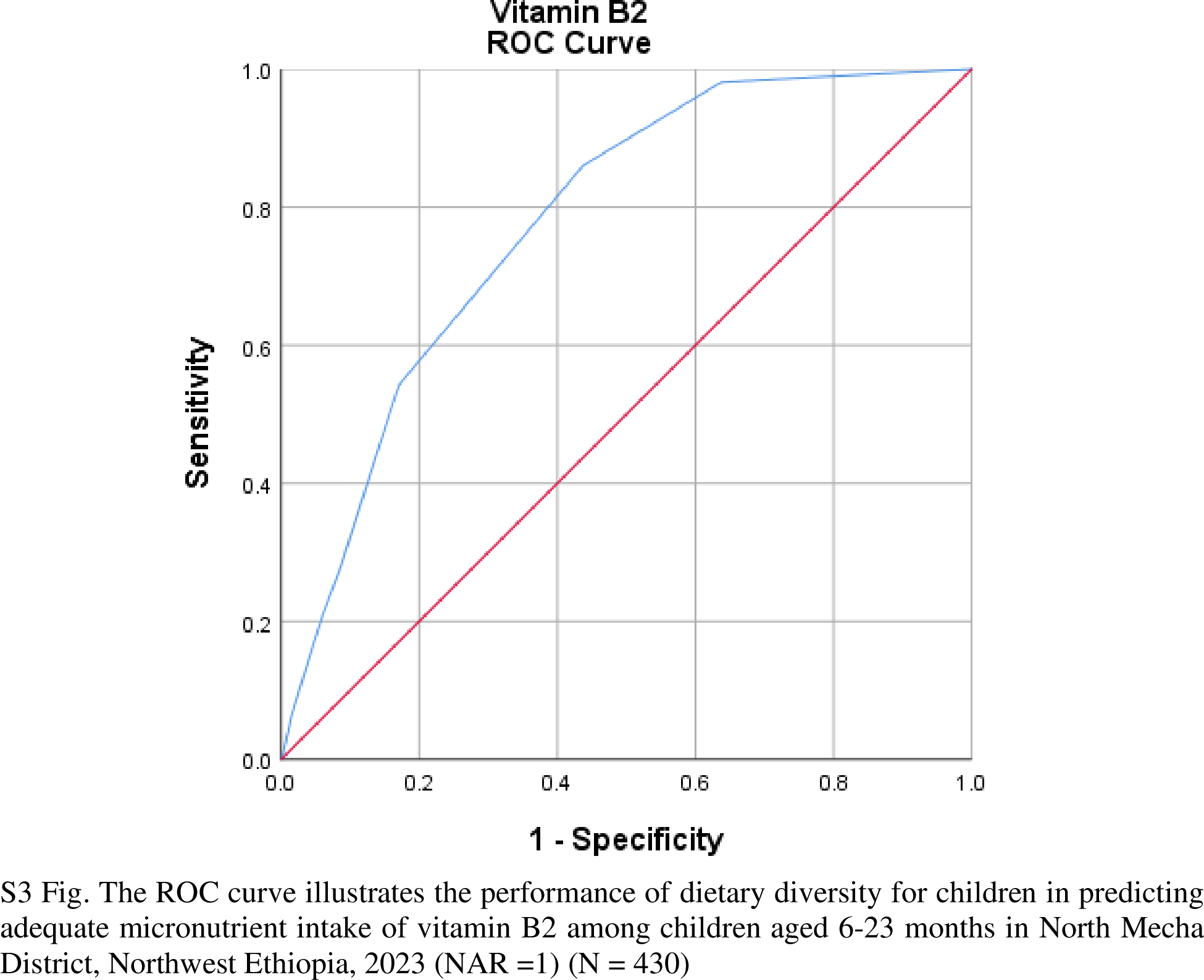

Supplement: S3 Fig — (TIF) [file pone.0334827.s003.tif]

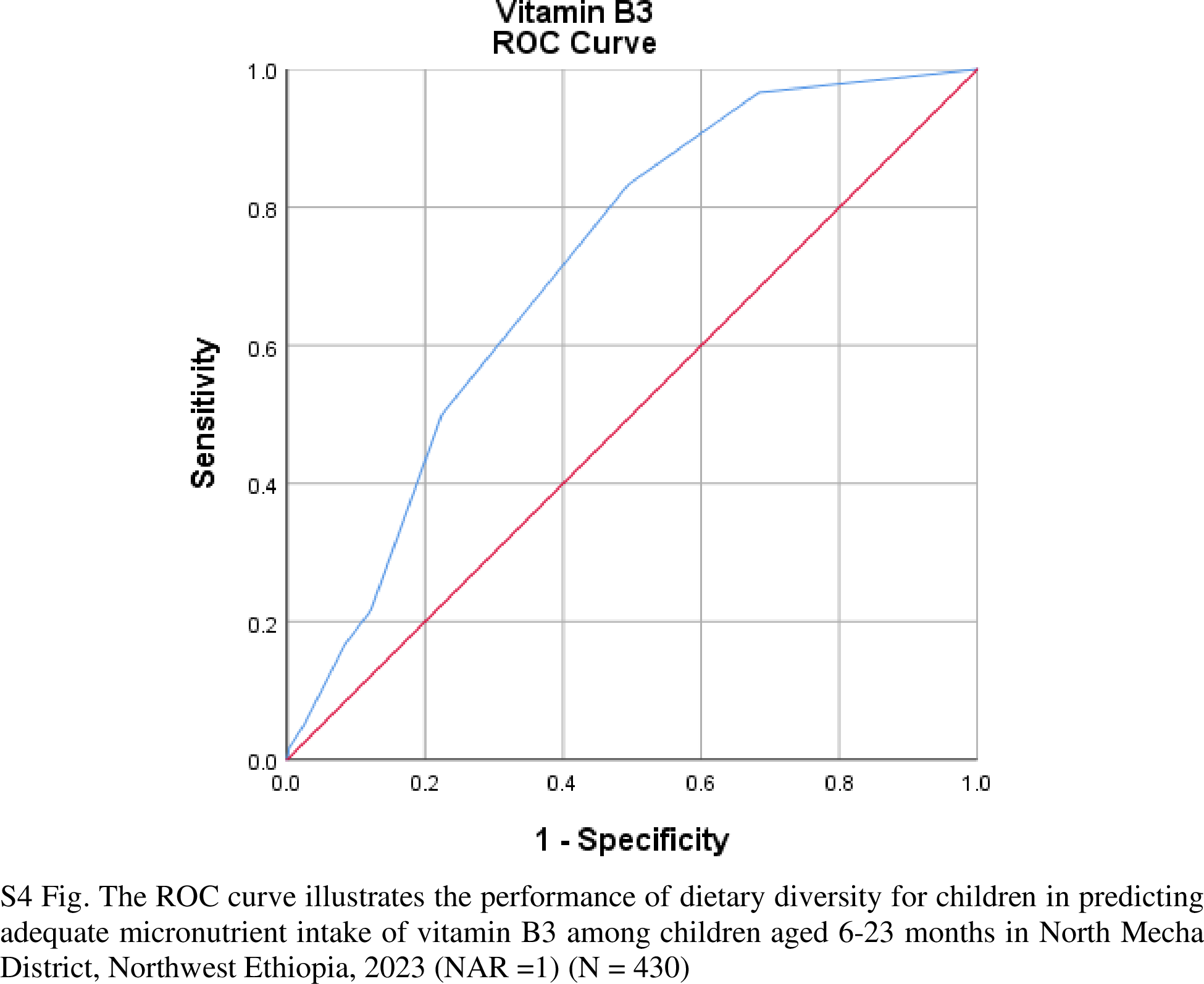

Supplement: S4 Fig — (TIF) [file pone.0334827.s004.tif]

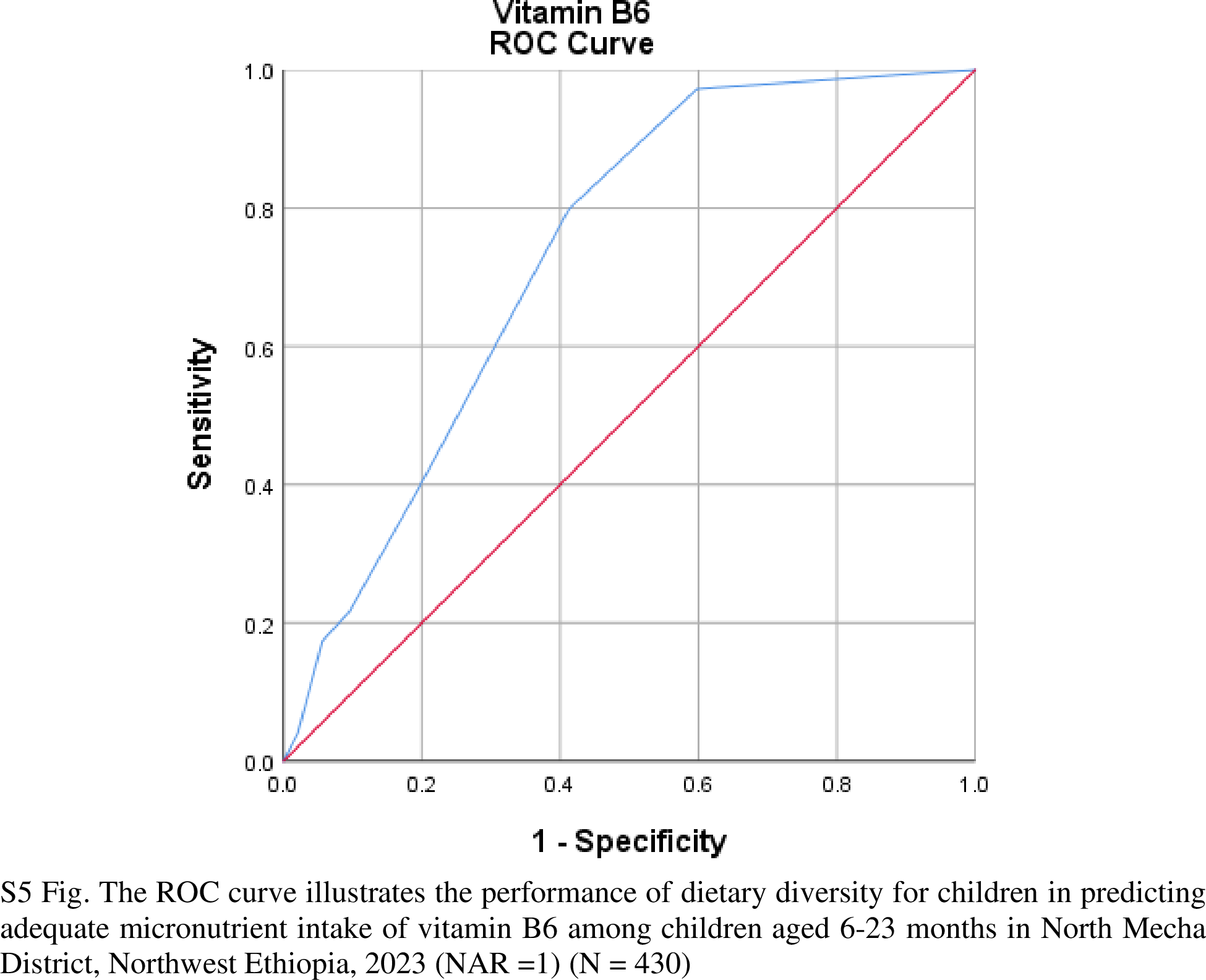

Supplement: S5 Fig — (TIF) [file pone.0334827.s005.tif]

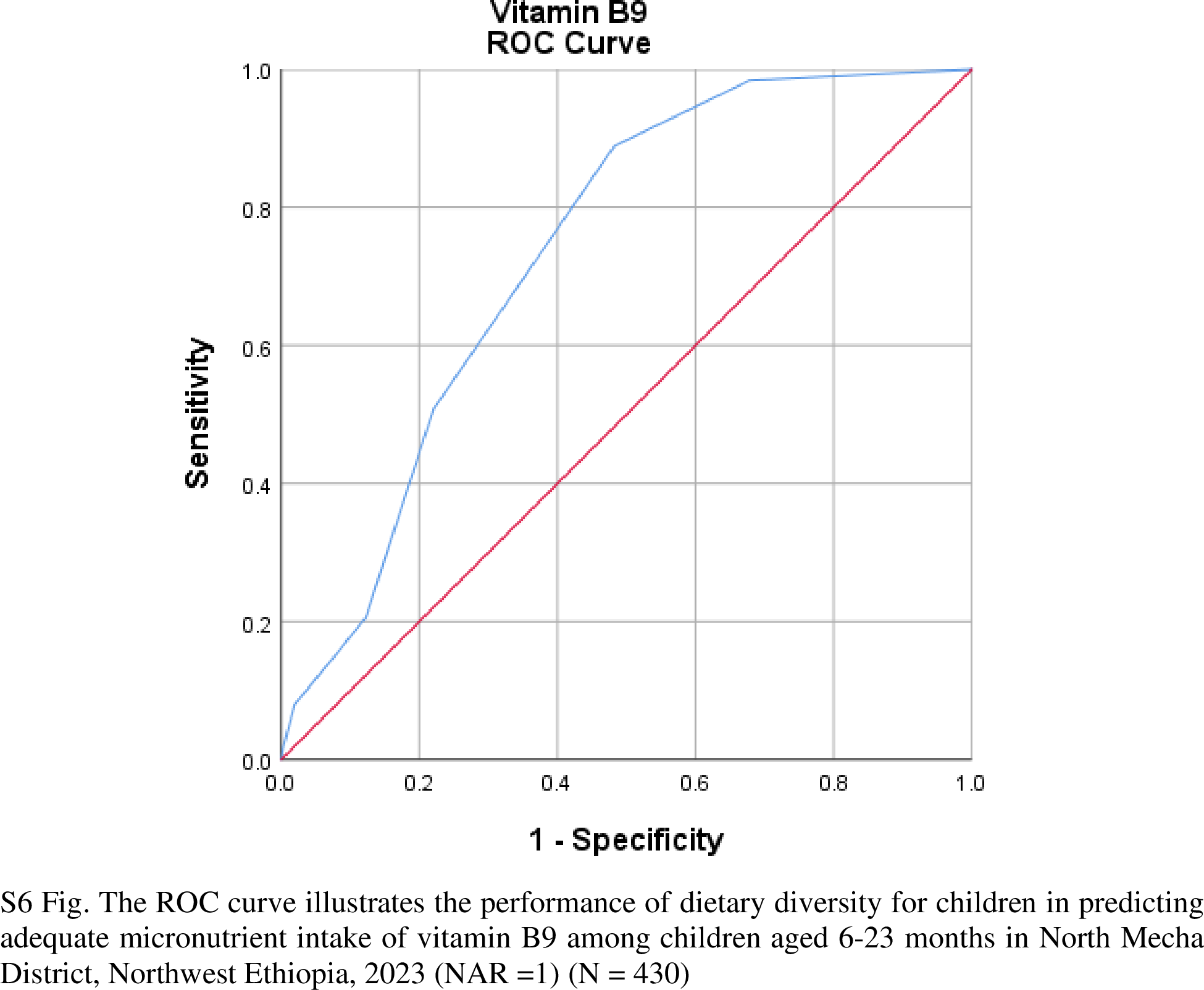

Supplement: S6 Fig — (TIF) [file pone.0334827.s006.tif]

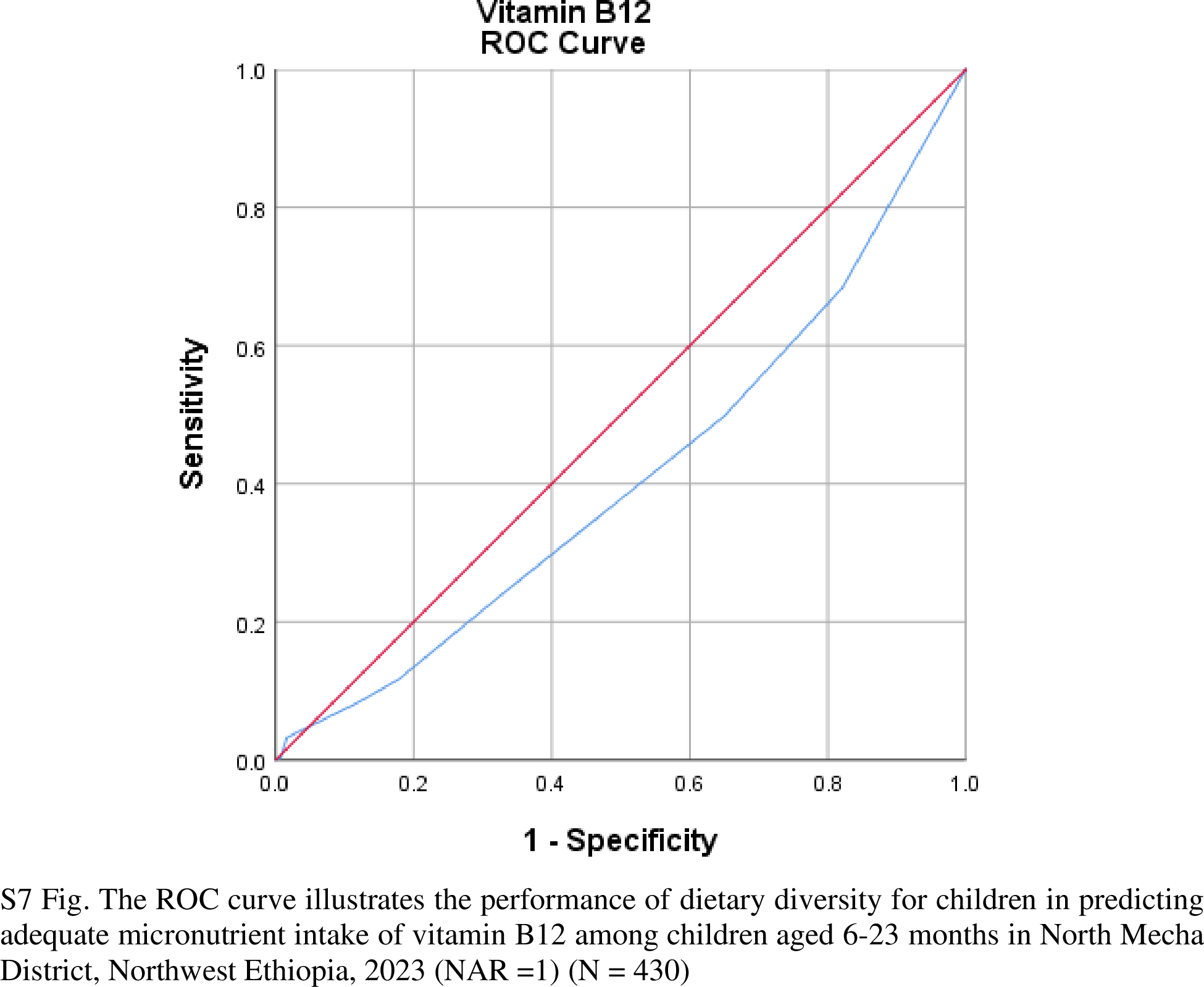

Supplement: S7 Fig — (TIF) [file pone.0334827.s007.tif]

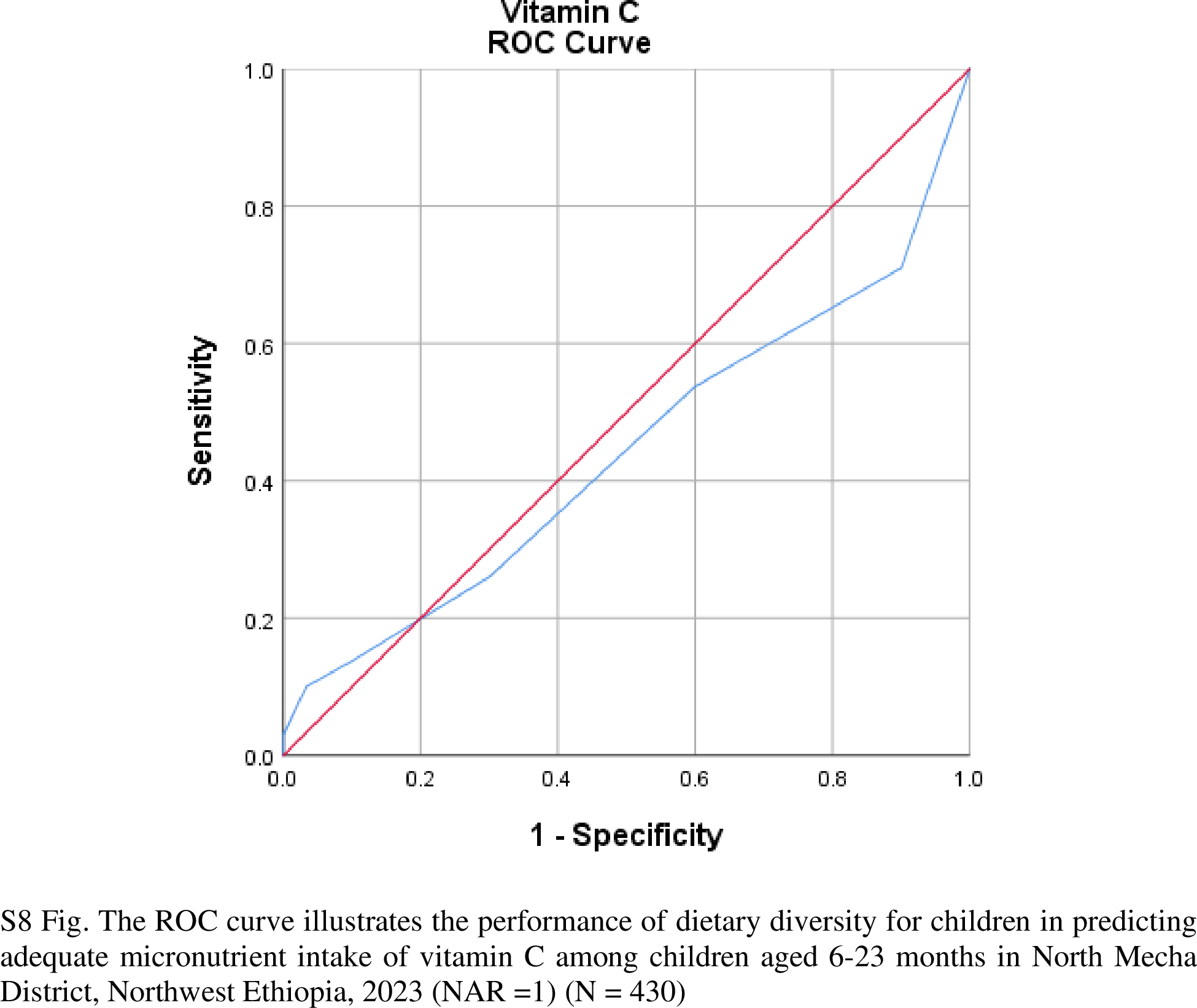

Supplement: S8 Fig — (TIF) [file pone.0334827.s008.tif]

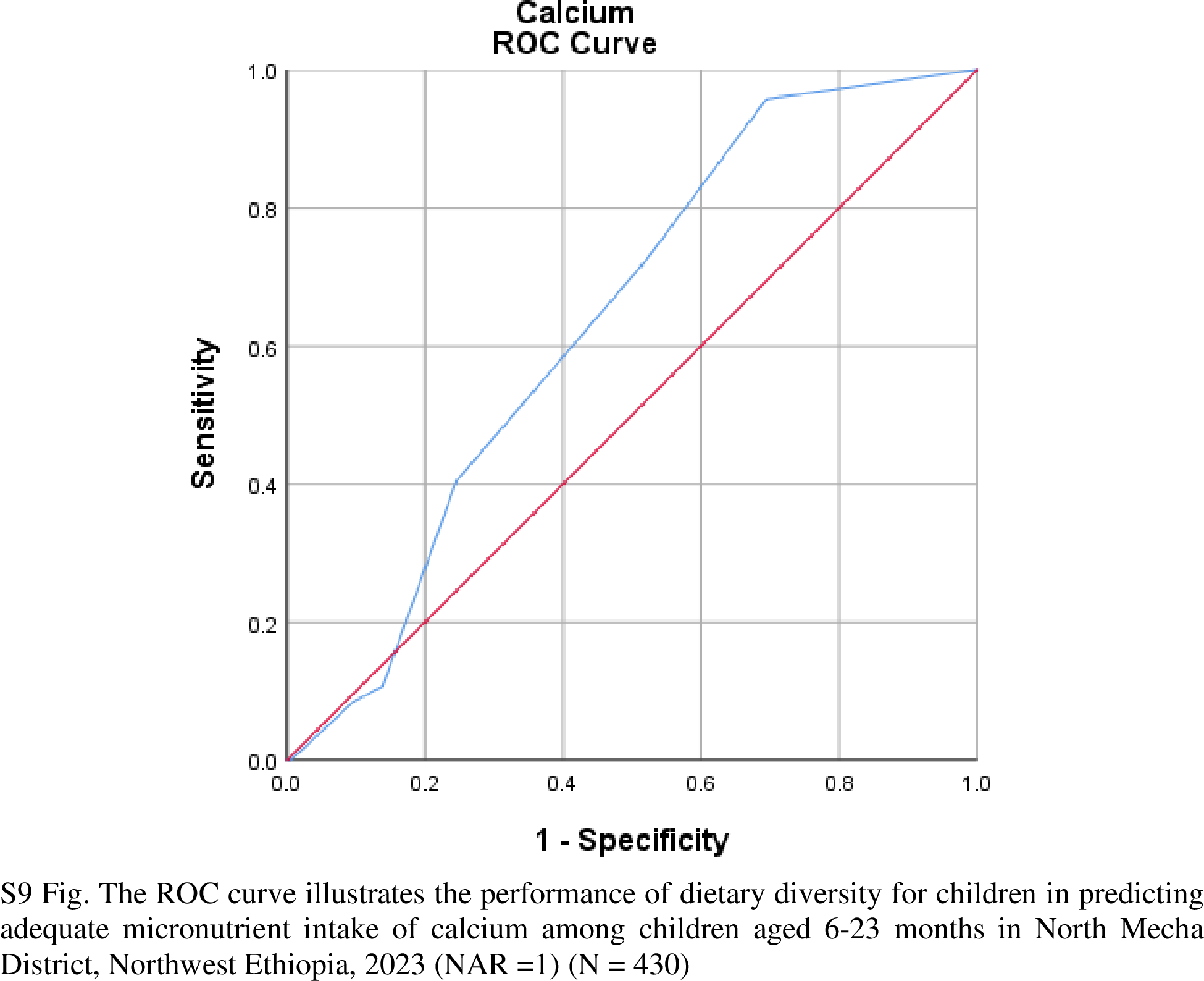

Supplement: S9 Fig — (TIF) [file pone.0334827.s009.tif]

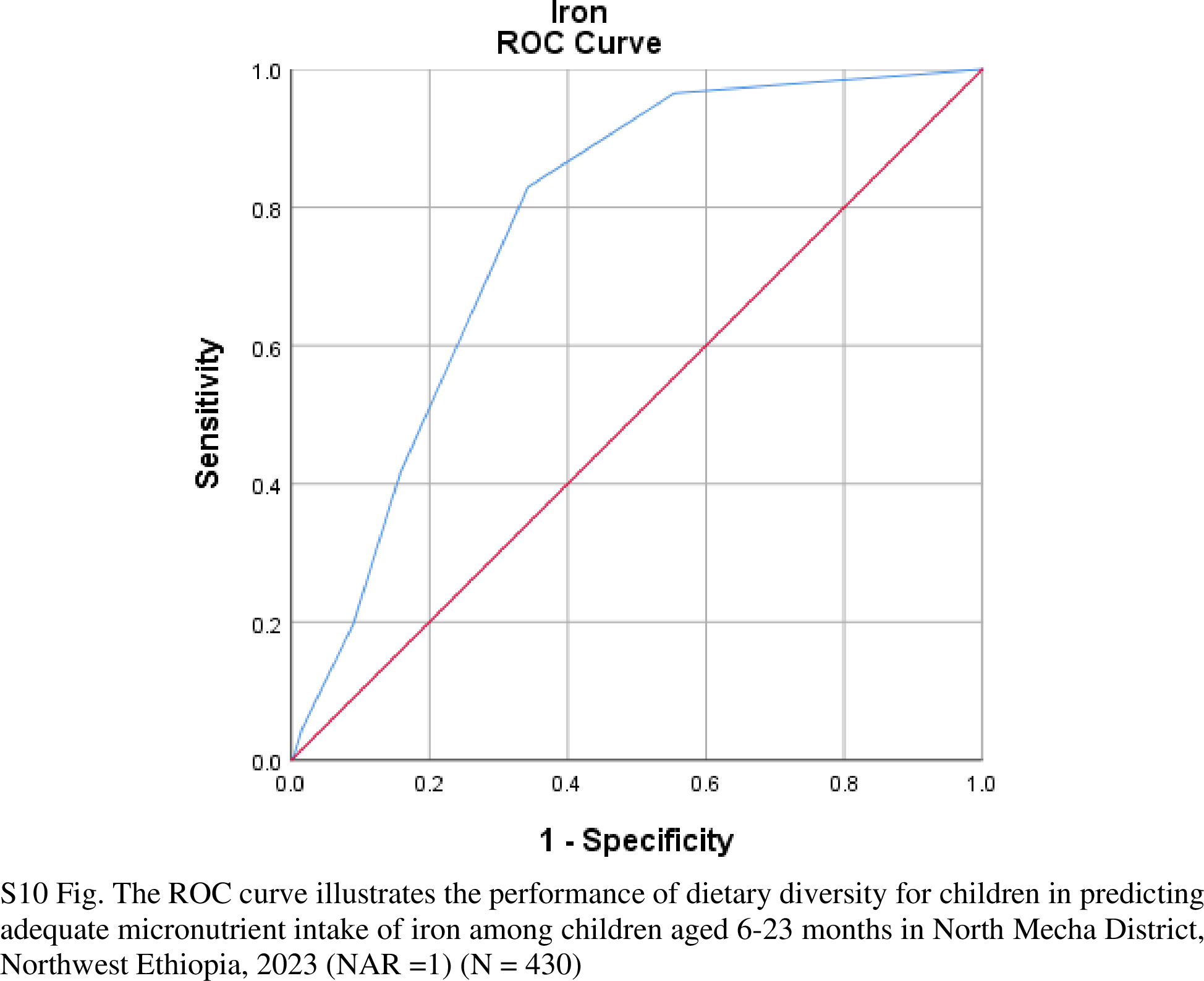

Supplement: S10 Fig — (TIF) [file pone.0334827.s010.tif]

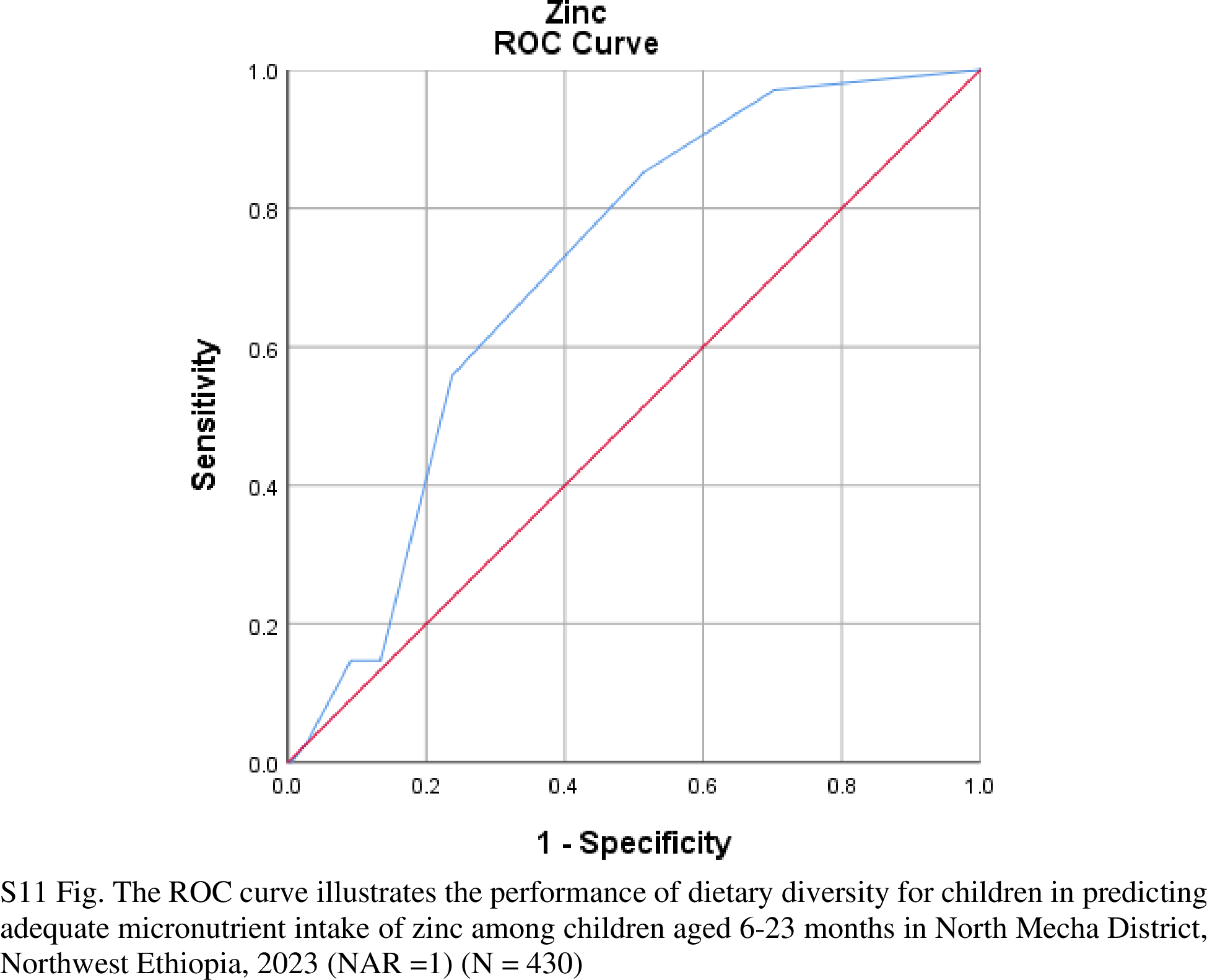

Supplement: S11 Fig — (TIF) [file pone.0334827.s011.tif]

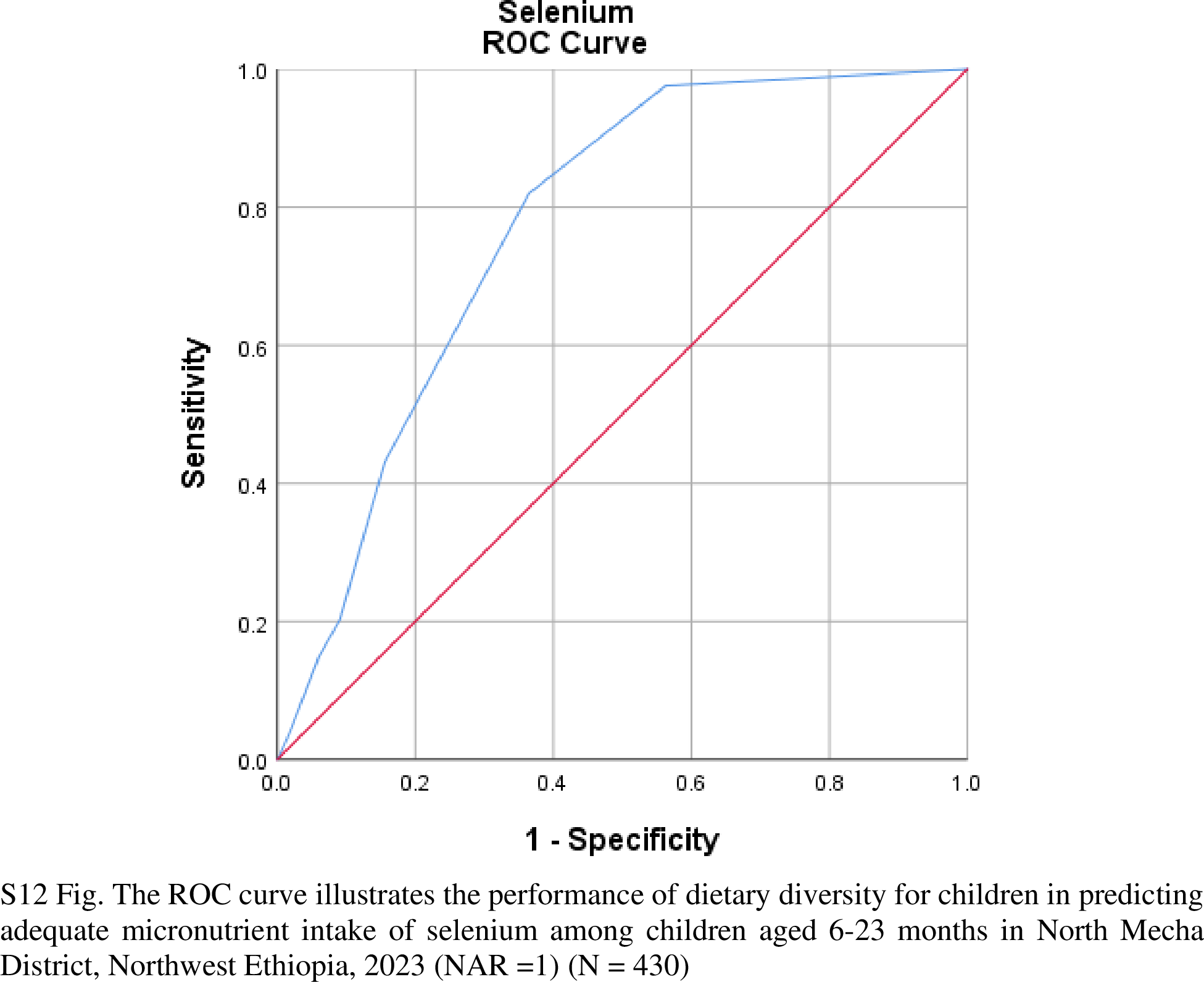

Supplement: S12 Fig — (TIF) [file pone.0334827.s012.tif]
